# Epigenetics, microRNA and Metabolic Syndrome: A Comprehensive Review

**DOI:** 10.3390/ijms22095047

**Published:** 2021-05-10

**Authors:** Farha Ramzan, Mark H. Vickers, Richard F. Mithen

**Affiliations:** Liggins Institute, The University of Auckland, Auckland 1023, New Zealand; m.vickers@auckland.ac.nz (M.H.V.); r.mithen@auckland.ac.nz (R.F.M.)

**Keywords:** DNA methylation, histone modification, epigenetic diet, microRNAs, prediabetes

## Abstract

Epigenetics refers to the DNA chemistry changes that result in the modification of gene transcription and translation independently of the underlying DNA coding sequence. Epigenetic modifications are reported to involve various molecular mechanisms, including classical epigenetic changes affecting DNA methylation and histone modifications and small RNA-mediated processes, particularly that of microRNAs. Epigenetic changes are reversible and are closely interconnected. They are recognised to play a critical role as mediators of gene regulation, and any alteration in these mechanisms has been identified to mediate various pathophysiological conditions. Moreover, genetic predisposition and environmental factors, including dietary alterations, lifestyle or metabolic status, are identified to interact with the human epigenome, highlighting the importance of epigenetic factors as underlying processes in the aetiology of various diseases such as MetS. This review will reflect on how both the classical and microRNA-regulated epigenetic changes are associated with the pathophysiology of metabolic syndrome. We will then focus on the various aspects of epigenetic-based strategies used to modify MetS outcomes, including epigenetic diet, epigenetic drugs, epigenome editing tools and miRNA-based therapies.

## 1. Introduction

The burden of non-communicable diseases (NCDs) or chronic diseases is increasing globally at an alarming rate and is currently the leading cause of mortality worldwide [1], with NCDs presently accounting for more than 70% of all deaths, most of which are preventable [2]. These diseases include cardiovascular diseases (CVD), chronic respiratory disease, cancer and type 2 diabetes (T2DM) [3]. The main risk factors underpinning these diseases involve aberrant changes in an individual’s metabolic and physiological profile that eventually result in the development of metabolic syndrome (MetS). MetS is defined as a multifactorial condition consisting of several inter-related anthropometric and biochemical features, including increased visceral adiposity, high fasting blood glucose, high blood pressure, low high-density lipoprotein-cholesterol (HDL-C) and high triglycerides, with further immune and vascular alterations [4]. The risk profile of MetS underlies the progressive development of diseases, including CVD and T2DM [5]. Complex interactions between environmental factors, including dietary and lifestyle, with the genetic and epigenetic makeup of an individual, are reported to be responsible for the pathophysiology of MetS [3,6,7]. However, the exact aetiology underpinning the development of MetS remains to be fully established [5].

Recent advances in the rapidly evolving field of epigenetics have revealed a complex network of shared interconnections between epigenetic machinery and human diseases [8,9,10,11]. Epigenetic modifications are reversible, differ across cell types and potentially lead to increased disease susceptibility by producing long-term changes in gene transcription [12,13]. Due to their key roles as mediators of gene regulation, epigenetic modifications may play a crucial role in developing pathological conditions [13]. Understanding the epigenetic machinery underlying MetS and how such epigenetic processes can provide utility in the development of diagnostic tools (i.e., biomarkers) and therapies is fundamental in improving the quality of life in individuals affected by MetS. Epigenetic changes can either be inherited or accumulated throughout a lifetime but, unlike genetic changes, are reversible and can therefore provide potential targets for MetS prevention and intervention [11]. Epigenetic patterns can also be altered under different developmental processes, dietary alterations, lifestyles or metabolic status, thus highlighting the importance of epigenetic factors as underlying processes in the aetiology of metabolic diseases [13,14,15].

This review will reflect on the classical epigenetic changes frequently associated with the pathophysiology of MetS, including DNA methylation and histone modifications. In addition to these mechanisms, we will then describe how miRNAs contribute to the epigenetic machinery of MetS by considering, firstly, how epigenetic mechanisms regulate miRNA expression and function and, secondly, how miRNAs reciprocally regulate the classical epigenetic mechanisms of DNA methylation and histone modifications. Finally, we will focus on epigenetic-based strategies used to modify MetS outcomes, including epigenetic diet, epigenetic drugs, epigenome editing tools and miRNA-based therapies.

## 2. The Classical Epigenetic Mechanisms in MetS

Epigenetics refers to the DNA chemistry changes that result in gene transcription modification and translation independently of the underlying DNA coding sequence. Epigenetic changes are increasingly thought to be of importance both in normal physiological processes and disease conditions [13]. The “classic” epigenetic mechanisms encompass DNA methylation and histone modifications, both of which can reduce or prevent RNA transcription and may be heritable [13,16,17]. Growing evidence has reported the function of classic epigenetic mechanisms in the regulation of gene expression [11,18,19]. However, the role of epigenetic marks in the development and progression of MetS is not clearly understood. Thus, exploration of epigenetic changes in the setting of the MetS may provide a deeper understanding of the molecular mechanisms and pathways involved. In this section, we focus on these classic epigenetic processes associated with the pathophysiology of MetS.

### 2.1. DNA Methylation

DNA methylation refers to a covalent and reversible transfer of a methyl group from S-adenosyl methionine (SAM) to the pyrimidine C5 position of the cytosine residues on genomic 5′-C-phosphate-G-3′ (CpG) dinucleotides, resulting in the formation of 5-methylcytosine (5-mC) [20]. A group of specific enzymes known as DNA methyltransferases (DNMTs) function as catalysers to inscribe the methylation marks on the genomic DNA. DNMTs are categorized into either maintenance DNMTs (DNMT1, DNMT2) that are involved in restoring the existing pattern of DNA methylation during cell replication, or de novo DNMTs (DNMT3a, DNMT3b and DNMT3L) responsible for catalysing new methylation of genomic DNA during embryonic development [21]. These DNMTs work in coordination to maintain a methylation pattern that supports a balanced transcriptional control over the genome. Methylation patterns within the promoter sequences of the DNA results in the downregulation of gene expression, while it has been reported that DNA methylation within the gene upregulates its expression. In contrast, the ten-eleven translocation (TET) family of enzymes, through their methylcytosine dioxygenase activity, assist in the removal of existing methylation marks by causing the conversion of 5-mC to 5-hydroxymethylcytosine (5-hmC) [20].

The association between aberrant DNA methylation and disease state has been studied across various disorders, including CVDs, autoimmune disorders and various cancers [22,23]. In recent years, epigenome-wide association studies (EWASs) have identified global and locus-specific epigenetic changes potentially involved in the pathophysiological mechanisms responsible for the development of MetS (Table 1) [24,25]. For example, an EWAS study performed in individuals with MetS demonstrated decreased methylation of CPT1A, a gene with a key role in regulating mitochondrial fatty acid oxidation (FAO), to be correlated with the increased metabolic risk and overall MetS phenotype [25]. In a recent EWAS study in African American adults, MetS was consistently associated with increased methylation in the ABCG1, a gene that encodes a protein in the ATP-binding cassette transporter family and is involved in intra- and extra-cellular signalling and lipid transport [24,26]. Additionally, it has been shown that marked differences exist in the prevalence of MetS and that of the DNA methylation patterns among middle-aged African Americans and white individuals diagnosed with MetS [25].

Further, with the acknowledged role of adipose tissue in the maintenance of metabolic homeostasis and altered adipogenesis associated with the MetS, studies have been undertaken to understand better the role of epigenetic processes in regulating the adipocyte function in the setting of such metabolic disorders [23,27,28]. A recent study by Daniel et al. [23] identified global DNA methylation of *LINE-1* to be associated with metabolic deterioration and glucose metabolism in visceral adipose tissue of individuals with and without MetS. Strong associations between methylation of specific genes involved in substrate metabolism (*LPL* and *PPARA*) and inflammation (*TNF*) with that of MetS status was also observed in these individuals [23]. Besides, it has been identified that alterations in dietary patterns could modulate the methylation patterns of key metabolic genes and hence their expression [29,30]. For example, it has been demonstrated that dietary total antioxidant capacity is positively correlated with the global DNA methylation of *LINE-1* levels in individuals with MetS after an 8-week hypocaloric diet [31]. Taken together, it is evident that DNA methylation has an important role to play in MetS development and progression; however, most of these studies only report associative effects and not cause–effect relationships. Of note, a potential limitation of observations to date is that many studies utilise analysis on whole tissues with mixed cell populations, thus, changes in methylation patterns observed may be due to tissue or cell population heterogeneity [32]. Furthermore, only small changes in methylation (1–10%) have been associated with complex diseases, and these observed differences in methylation profiles could arise as a result of variations in cell populations within the same tissue [33]. However, the functional consequences of such small absolute changes in methylation patterns in the setting of MetS cannot be discounted.

**Table 1:** Human studies showing associations between aberrant DNA methylation and features of metabolic health.

Disease	Differentially Methylated Genes	Sample Type	Ref
MetS	CPT1A	CD4+ T cells	[34]
MetS	ABCG1	Blood (buffy coat)	[26]
MetS	LINE-1	Visceral adipose tissue	[23]
T2DM	PPARGC1A	Pancreatic islets	[35]
T2DM	TXNIP	Leucocytes	[36]
Hypercholesterolemia	TNNTI	Blood	[37]

### 2.2. Histone Modifications

Classical epigenetic mechanisms also involve post-translational modifications of histone proteins by specialized histone-modifying enzymes, resulting in changes to chromatin architecture and gene expression regulation [38]. There are several post-translational modifications of histone proteins, with acetylation, phosphorylation, methylation and ubiquitination of histones being the most commonly observed [39]. Methylation of histones could result in either an either an increase or decrease in gene expression. For example, methylation of histone H3 at lysines 9, 27, and 36 would reduce gene expression, while methylation of histone H3 at lysines 4 and 79 and methylation of histone H4 at lysine 20 would generally result in an increase in proximal gene expression [40]. The dynamics underlying these changes are mediated through histone methyl transferases that place methyl groups and histone demethylases that remove methylation [39]. Similarly, acetylation of lysines on histones H3 and H4 is associated with increased transcription of nearby genes. Acetyl groups are placed by histone acetyl transferases and removed by histone deacetylases (HDAC). Histone phosphorylation is less well studied but is suggested to increase gene expression in combination with H3K56ac [9]. Histone variants such as H2A.Z and H3.3 have been demonstrated to play specific roles in regulating chromatin structure and function by influencing transcription of nearby genes [15].

Alterations in histone modifications are identified as essential components of epigenetic networks, controlling energy homeostasis and altering adipocyte thermogenesis, thereby contributing to the pathogenesis of MetS (Table 2) [28,41]. For example, a study involving mice deficient in histone demethylase, HDM2a, an enzyme responsible for H3K9 demethylation, reported the development of adult-onset obesity, hypertriglyceridemia and hypercholesterolemia, as well as insulin resistance (IR), in the deficient mice as compared to the wild type [41]. Additionally, histone modifications are also reported to interact with pathways related to the development of IR and inflammation, important hallmarks of MetS [42]. Consistent with this notion, IR has been shown to positively correlate with *HDAC3* activity and HDAC3 mRNA levels in peripheral blood mononuclear cells of T2DM patients [43]. Moreover, sirtuins (SIRT, a class of HDACs) are reported to act as metabolic regulators of glucose homeostasis and IR-associated inflammation [44,45]. A lack of SIRT1-, SIRT2- and SIRT6-dependent deacetylation and activation of specific adipose gene programs have been shown to contribute to the development of metabolic disorders, including obesity andT2DM [46,47], thus further suggesting a role for histone modifications in the aetiology of metabolic disorders.

## 3. Epigenetics and miRNAs in MetS

While growing evidence has demonstrated an important role of classical epigenetic mechanisms, including DNA methylation and histone modifications, it is important also to consider factors outside of the nucleotide sequence that could affect gene expression. These factors alter the gene expression by affecting underlying mechanisms responsible for how transcription factors and other proteins bind to DNA, thus changing gene expression, which affects metabolism. In the context of such factors, there is increasing recognition of the role of small RNA molecules, particularly microRNA (miRNA), in the epigenetic regulation of gene expression [13,19,51].

miRNAs are evolutionary conserved, short non-coding RNA molecules of size ~18–24 nucleotides and are known to be involved in post-transcriptional and transcriptional regulation of gene expression and protein synthesis [52]. More than 2500 mature miRNA species have been discovered in the human genome [53]. It has been reported that >60% of protein-coding genes in the human genome are targeted by miRNAs [54], with a single miRNA being able to target and regulate several thousand mRNAs [55]. miRNAs function by binding to complementary sequences on the 3′-untranslated region (3′UTR) of the target messenger RNA (mRNA), thereby decreasing its stability and translation efficiency [56]. Dysregulation in the expression of miRNAs has been shown to modulate pathological pathways involved in the development of various diseases [57], such as T2DM [58,59], cancer [60,61] and CVD [62,63], and therefore have utility as potential biomarkers or diagnostic tools for both normal physiological and disease states [64].

Interestingly, the interaction of miRNAs with their gene targets is dynamic and dependent on various factors, such as their subcellular location, including mitochondria, endoplasmic reticulum and multivesicular bodies, their abundance, target mRNAs and the affinity of the miRNA–mRNA interaction [65]. Emerging studies to date have not only established the role of miRNAs in key physiological processes, including cell proliferation, DNA repair, cellular differentiation, insulin secretion, aging, metabolism and apoptosis of the host cell [66,67], but have shown them to be secreted into extracellular fluids and transported to target cells via vesicular bodies, such as exosomes, microvesicles or by binding to Argonaute (Ago) proteins (25) acting as the cell to cell mediators, thereby regulating their gene expression in a paracrine or endocrine manner [68,69].

In addition to their post-transcriptional functions, miRNAs are also reported to be involved in transcriptional gene regulation by binding to specific sequences of epigenetic events on DNA [70]. For example, miRNAs control the expression of various epigenetic regulators, such as DNA methyltransferases (DNMTs) and histone deacetylases (HDACs) [71]. Similarly, DNA methylation and histone modifications can regulate the expression of some miRNAs. Taken together, miRNAs and epigenetic regulators cooperate to modulate the expression of mutual targets by forming a feedback loop. Therefore, although not strictly considered epigenetic factors, miRNAs contribute to gene expression modulation through epigenetic mechanisms. Any disruption of this complex regulation may participate in the development of different diseases, including MetS.

### 3.1. Epigenetically-Regulated miRNAs in MetS

Previous studies investigating the pathophysiology of MetS have revealed a complex network of reciprocal interconnections between those of miRNAs and the classic epigenetic machinery of DNA methylation and histone modifications [19,51,72]. In addition to regulating structural genes by the classical mechanisms, they have an important role in regulating miRNA expression [51,73]. DNA methylation regulates miRNA transcription either by hyper- or hypo-methylation of the promoter regions of miRNA genes [74]. Indeed, around 50% of the miRNAs are associated with CpG islands [75], and methylation of these sites on miRNA promoters can result in their modified expression [73,76]. For instance, it has been observed that pancreatic-β cells of individuals with T2DM have a cluster of miRNAs that are epigenetically regulated by hypermethylation of the DLK1–MEG3 locus on chromosome 14q32 [77]. A study by Wang et al. [78] examining expression levels and DNA methylation of miR-375, one of the most abundant miRNAs in the human pancreatic β cells, identified hypermethylation of its promoter and downregulated expression in the plasma of individuals with impaired glucose tolerance compared to those with normal glucose tolerance. Further, methylation of CpG sites located in the coding regions of miR-1203, miR-412 and miR-216a is responsible for their differential expression in obese children’s peripheral blood compared to non-obese children [79].

Epigenetic regulation of miRNA expression cannot be represented by DNA methylation alone but also involves histone post-translational modifications [80,81]. While the impact of alterations in the histone modifications on miRNA expression has not been extensively investigated in MetS per se, limited studies to date have implicated a regulatory role of histone-modified epigenetic regulations on miRNA expression during various pathophysiological mechanisms responsible for MetS development. Among these modifications, chromatin structures have been associated with the biogenesis and post-transcriptional regulation of miRNAs [82]. Several miRNAs related to metabolism are shown to be regulated by a repressive chromatin structure involving H3K27me3 mediated by an epigenetic regulator enhancer of zester homolog 2 (EZH2) [83,84]. Expression of miR-101-3p, a pancreatic islet-enriched miRNA with a role in insulin secretion and β cell functioning [85], is demonstrated to be regulated by H3K27me3 modification in EZH2. Moreover, epigenetically regulated alterations in miRNAs induced by suboptimal maternal nutrition or endocrine factors are also reported to be responsible for altered gene expression and to promote offspring and adult MetS phenotypes [86,87]. Altogether, these studies provide evidence for a functional link between classical epigenetic regulations of miRNA underpinning pathophysiological processes characterised as causal factors in the development of MetS.

### 3.2. miRNA-Induced Epigenetic Regulation in MetS

miRNAs are reported to reciprocally regulate the classical epigenetic processes and play an essential role in the pathophysiological mechanisms of various diseases, such as cancer and T2DM [88]. Numerous studies provide insights into miRNA contributing to the pathophysiology of metabolic-related diseases through the regulation of DNA methylation [78,89]. miRNAs are shown to regulate the expression of key DNA methyltransferases (DNMTs), including DNMT3a and DNMT3b and methylation-related proteins involved in de novo methylation [90]. For instance, miR-148, an identified circulatory biomarker of MetS [91], targets DNMT3b at the penultimate exon of their coding regions [92]. Similarly, members of the miR-29 family are shown to target DNMT3a and DNMT3b [87] directly. Dysregulation in the expression levels of miR-29 has been linked to impaired metabolic function, including altered insulin sensitivity and glucose metabolism, thus contributing to the pathogenesis of MetS [93,94]. In addition, a study involving a model of obesity in the mouse identified hepatic miR-29b to regulate DNMT3a and the hormone-encoding gene energy homeostasis-associated (*Enho*) in modulating insulin sensitivity [93].

It is well known that miRNA-mediated epigenetic changes induce obesity-associated adipose tissue inflammation, a significant factor responsible for developing IR and T2DM [95]. For example, it has been shown that obese adipocyte-derived exosomes have an increased expression of miR-29a. When transferred into adipocytes, myocytes and hepatocytes, these exosomes could result in IR both in in vitro and in vivo models [96]. Furthermore, SIRT1, a class-III histone deacetylase with an essential role in inflammation and metabolic homeostasis, has been identified to be regulated by miRNAs in the adipose tissue of individuals with obesity [44,97]. For instance, miR-377, an important regulator of adipogenesis, has been shown to target the 3’-UTR of SIRT1 mRNA directly, and downregulate its protein abundance, thereby promoting obesity-induced inflammation and IR [98]. Decreased insulin sensitivity, an important hallmark of MetS, has also been reported to be affected by miRNA-induced epigenetic alterations, partly through suppression of SIRT1 [47]. For example, miR-221, an miRNA known to affect adipocyte differentiation, metabolic homeostasis and insulin signalling [99], has been shown to promote adipose tissue inflammation by negatively regulating SIRT1 and decreasing adipose tissue insulin sensitivity [100]. miRNAs are also reported to suppress the differentiation of preadipocytes in a mouse model of obesity by involving the interaction of miR-138-5p with the 3’UTR of EZH2 [101]. Additionally, miR-22-3p acts as an inhibitor of adipogenic differentiation by suppressing histone deacetylase-6 (HDAC6) in human adipose tissue-derived mesenchymal stem cells [102]. Considering the evidence to date, these findings provide a framework for miRNAs in regulating the epigenetic machinery underlying the pathophysiological mechanisms linked to MetS development and progression.

## 4. Epigenetic Strategies for MetS Prevention/Reversal

The epigenome is in constant feedback with both the genotype and phenotype of an organism, and it has a profound effect on the pathophysiology of MetS [42]. Moreover, it has been reported that environmental factors, including diet and nutrition, can trigger the change in epigenetic regulation responsible for the pathophysiology of MetS. Therefore, developing interventions to modify the epigenetic alterations would enable ameliorating MetS and related metabolic disorders. These epigenetic-based strategies would include pharmacological interventions, epigenetic-based diets, editing tools for modifying the epigenome and miRNA-based therapeutics and diagnostic tools.

### 4.1. Epigenetic–Pharmacological Interventions

Epigenetic modifications are potentially reversible, and therefore pharmacological interventions targeted at these modifications hold great potential for the prevention, diagnosis, treatment and prognosis of MetS [22]. Drugs targeting epigenetic changes are termed either “epigenetic drugs” or “epidrugs”. Possible epidrugs would involve therapies that can alter DNA methylation, histone/chromatin modifications or miRNA expression [103]. Epidrugs mainly act as inhibitors for most of their targets but can also potentially activate them. Currently, several drugs targeting the enzymes necessary for undergoing modifications and nucleoside and non-nucleoside analogues of epigenetic changes are undergoing preclinical and clinical trials [14].

Since aberrant methylation of metabolic gene promoters and overexpression of DNMTs has been established as one of the major key players in the pathophysiology of MetS, demethylating agents such as DNMT inhibitors appear to offer promise as potential therapeutic targets for MetS. DNMT inhibitors function by blocking the key methylation enzymes such as DNMT1, DNMT3a and DNMT3b, which subsequently blocks DNA methylation. For instance, 5-azacitidine, a structural analogue of cytosine, when incorporated into DNA, is methylated by covalently bound DNMT3a, eventually inhibiting the enzyme activity [104]. A study by You et al. [105] utilizing insulin-resistant 3T3-L1 adipocytes identified an increased percentage of insulin-stimulated glucose uptake upon treatment with 5-azacitidine [105]. Another study employing a rat model of obesity observed that DNA hypermethylation was associated with reduced insulin sensitivity [106]. Using insulin-resistant rat hepatocytes, the authors observed that treatment with 5-azacitidine could restore insulin sensitivity [106].

One other class of epidrugs would involve modulators of histone acetylation, i.e., inhibitors of histone acetyltransferase (HATi) and inhibitors or activators of histone deacetylase (HDAC) [107]. For instance, tannic acid (TA), a plant-derived hydrolysable tannin polyphenol and a novel HATi, is reported to potentially attenuate lipid accumulation and ameliorate the development of non-alcoholic fatty liver diseases via inhibition of HAT activity [108]. Evidence from several preclinical and clinical trials suggests that other HDACis, including valproic acid (VPA), sodium phenylbutyrate (PBA) and trichostatin A (TSA), could have beneficial roles in reducing fat accumulation, IR, inflammation and gluconeogenesis, thus exerting anti-diabetic effects [109,110,111,112,113]. A different category of epidrugs would include regulators of SIRTs [114]. For example, fluvastatin, an inhibitor of 3-hydroxy-3-methylglutaryl-coenzyme A reductase (HMG CoA), is reported to act as the activator of SIRT6 in liver cell models via phosphorylation of the AMPKα and SREBP-1 pathway, thereby maintaining cholesterol regulation [115]. Apart from acting as an activator, small molecules, such as 2,4-dioxo-N-(4-(pyridin-3-yloxy) phenyl)-1,2,3,4-tetrahydroquinazoline-6-sulfonamide, are identified to act as Sirt6 inhibitors in mice models of T2DM [116]. This molecule was identified to improve glucose tolerance in the mice by increasing the glucose transporters GLUT1 and GLUT1 and reducing insulin, triglycerides and cholesterol levels.

Epidrugs have the potential to provide novel therapeutic agents for various diseases, including MetS. However, there are still challenges remaining for implementing epigenetic-based approaches in the clinic [14]. In particular, to achieve the promise of reversing epigenetic alterations by genome-wide-acting epidrugs, a comprehensive understanding of their mechanism of action and specificity in relation to a specific disease and tissue is required [117].

### 4.2. Epigenetic Diets

Dietary factors play a significant role in human health, with dietary bioactive compounds reported to act as key mediators of epigenetic reprogramming [15,118]. The diets or dietary compounds identified to mediate metabolic programming through epigenetic modifications are termed “epigenetic diets”. Epigenetic diets not only modify the classic epigenetic mechanisms of DNA methylation and histone modifications, but they have also been identified to interact with miRNAs and are therefore involved in the dynamic regulation of gene expression, controlling cellular phenotypes linked to both the prevention and progression of disease phenotypes [119].

Growing evidence involving human clinical and dietary intervention studies supports the importance of individual nutrients, whole foods and dietary patterns in preventing and managing metabolic disorders by altering the epigenetic modifications [118,119]. Among such diets, long-chain omega-3 polyunsaturated fatty acids (*n*-3 PUFAs) have been shown to have anti-obesity effects by modulating epigenetic mechanisms responsible for the underlying pathophysiology, including lipid metabolism adipokine regulation, adipose tissue inflammation and adipogenesis. Several animal and human studies have also reported consumption of *n*-3 PUFAs to modulate the expression of miRNAs involved in key metabolic pathways, such as lipid metabolism and inflammation [120]. Likewise, Mediterranean diets, due to their abundant source of phytochemicals, are reported to have positive health effects by modulating the epigenomic mechanisms regulating processes related to metabolic homeostasis and pathophysiological pathways involved in metabolic disorders [121,122]. An example of one of the essential components found in the Mediterranean diet would be that of sulforaphane, an isothiocyanate known to reduce hepatic glucose production and improve glucose control in individuals with obesity and T2DM [123,124]. Based on the molecular mechanism of action of sulforaphane, epigenetic control of histone deacetylase (HDAC) and DNA methylation activity could be a possible mechanism underlying the observed changes in glucose control [125]. Both acute and long-term Mediterranean dietary patterns have also been shown to modulate miRNA expression associated with the pathogenesis of MetS, such as inflammatory gene regulation, atherogenic mechanisms and adipogenesis [126,127,128,129]. For example, a study analysing the expression of miRNAs in response to 8 weeks of intake of a hypocaloric Mediterranean diet in individuals with MetS reported an altered expression of miRNAs (decreased miR-155 and increased let-7b) that are involved in the pathogenesis of CVD [127].

Another example of dietary-derived HATi would involve curcumin, a polyphenolic compound in turmeric. Curcumin is reported to decrease hyperglycaemia-induced cytokine production in monocytes via reducing HAT activity [15,130]. Among activators of histone deacetylase, resveratrol, a small polyphenolic compound, has been identified to improve hepatic gluconeogenesis under IR conditions by acting via translocation of HDAC4 from the nucleus to the cytoplasm, thereby modulating the energy metabolism pathway [131,132]. Subsequently, several human clinical trials have shown positive health effects of resveratrol supplementation in individuals with obesity, NAFLD or T2DM [133,134,135]. In addition, resveratrol supplementation is also identified to modulate the expression of inflammation-related miRNAs [136,137]. For instance, a study by Carneriro et al. reported that a daily intake of grape extract and resveratrol-containing supplement resulted in the upregulation of miR-21, miR-181b, miR-663 and miR-30c and the downregulation of miR-155 in peripheral blood mononuclear cells of T2DM and hypertensive patients with coronary artery disease [138]. Moreover, the authors also showed an inverse relationship between the upregulated miRNAs and inflammatory cytokine gene expression [138].

Additionally, natural phenolic compounds, including catechin, epicatechin and their oligomers proanthocyanidin and epigallocatechin-3-gallate (EGCG), are considered biological modulators of MetS [30,139]. In both human and animal models of obesity, catechins are shown to attenuate dyslipidaemia and IR and reduce concentrations of inflammatory cytokines [140,141,142]. It has been reported that catechins mediate these effects, in part, by modulating epigenetic mechanisms such as DNMT inhibition, increasing HDAC activity or by inhibition of HAT activity [143,144]. Further, polyphenols are also reported to modulate miRNA expression involved in regulating genes and pathways underlying MetS [145]. For example, using a high-fat diet-fed mice model, it was shown that following intake of green tea for 12 weeks, mice showed a decrease in adipose miR-335 with an increase in energy expenditure and a reduction in adipose tissue inflammation and IR-associated gene expression [146].

Another class of epigenetic diets would include diets that are involved in the modulation of one-carbon metabolism. One-carbon metabolism comprises a complex network of pathways involved in transferring and utilising one-carbon units necessary for nucleic acid biosynthesis, amino acid metabolism and methylation processes [147,148]. Growing evidence has suggested important links of one-carbon metabolism with insulin sensitivity pathways, fat deposition and energy homeostasis [149,150,151]. These diets would involve nutrients containing methyl donors such as choline, betaine and folate that serve functional roles across the body through their metabolic, epigenetic and immunomodulatory properties [152,153,154]. Based on the fact that one-carbon metabolism involves methyl donors for epigenetic reactions and that miRNA has an important role to play in the modulation of these mechanisms, this might suggest a bi-directional association between one-carbon metabolites and miRNA profiles [155,156].

Collectively, epigenetic diets may act as important adjuncts to the management of MetS. Supplementation of epigenetic diets would help enhance metabolic homeostasis by ameliorating processes involved in the pathophysiology of MetS, including inflammation, obesity, glucose intolerance and insulin insensitivity. Understanding the molecular targets of epigenetic diets in relation to maintaining metabolic homeostasis and the pathophysiology of metabolic disorders could help discover novel and effective therapeutic targets.

### 4.3. Epigenome Editing Tools

Changes in epigenetic patterns can ultimately alter an entire metabolic pathway, and these epigenetically disturbed pathways could serve as key targets for the treatment of various metabolic diseases, including MetS. Given the reversible nature of epigenetic modifications, developing tools to regulate gene expression by modifying these epigenetic states could be of significant importance [157,158]. The widely used epigenetic editing tools include the zinc finger proteins (ZFPs), transcription activator-like effectors (TALEs) and Clustered Regularly Interspaced Short Palindromic Repeats (CRISPR)-deactivated nuclease CRISPR-associated Cas9 protein system (dCas9) [159,160]. ZFPs and TALEs are modular DNA-binding proteins that form specific interactions between amino acid side chains of the DNA-binding domain and the nucleotides of target DNA sequences [157,161]. In contrast, the CRISPR-dCas9 targets DNA by enabling RNA:DNA base pair complementarity [160]. These tools, combined with epigenetic enzymes such as DNA methyltransferases, histone lysine/methyltransferases and histone deacetylases/demethylases, enable the reversibility of epigenetic modifications in a gene-specific manner.

The use of epigenetic editing tools for MetS is still in its infancy, although there is growing interest in using these tools for other related metabolic diseases, such as T2DM [162]. Since the loss of insulin-secreting β-cells is characteristic of the diabetes pathophysiology and because these cells do not proliferate readily, expanding the source of these cells remains a challenge. To enhance the rapid proliferation of human β-cells, a study by Ou et al. [163] utilized epigenetic editing tools to promote the proliferation of human β-cells in a diabetic, immune-compromised mice model. The authors used in situ human β-cells to target demethylation of imprinting control region 2 (ICR2) using a transcription activator-like effector protein fused to the catalytic domain of TET1 (ICR2-TET1) and repressed expression of p57 (cell cycle inhibitor). Further, transplantation of these epigenetically edited β-cells into diabetic immune-compromised mice reduced blood glucose levels. Another example includes epigenetically modified expression of *Pcsk9*, a gene responsible for regulating circulating cholesterol levels [157]. Systemic administration of a dual-vector adeno-associated viral 8 system (AAV8) system expressing dCas9 fused to the Krüppel-associated box epigenetic repressor motif (dCas9^KRAB^) and a *Pcsk9*-targeting guide RNA (gRNA) in the liver of adult mice resulted in reductions in the levels of both circulating Pcsk9 and cholesterol. Taken together, these studies establish the potential of epigenetic editing tools for dissecting gene regulation mechanisms, understanding the pathophysiological mechanism of MetS and modulating gene expression for therapeutic applications.

### 4.4. miRNA-Based Therapy and Diagnostics

Growing evidence has established dysregulated miRNA expression as a molecular signature in MetS. Like classical epigenetic mechanisms, targeting miRNAs is considered a potential method for developing novel therapeutic targets for MetS. miRNA-based therapeutics rely on inhibition of the upregulated miRNAs (antagomiRs) or the restoration of downregulated miRNAs (mimics). Moreover, because miRNAs are stably present in human bio-fluids and are disease- and tissue-specific, quantifying their abundance in blood samples has gained significant attention given their potential utility as biomarkers of MetS diagnosis, prognosis and response to treatment.

#### 4.4.1. miRNA Therapeutics

Considering that miRNAs are involved in regulating multiple pathophysiological pathways with relevance to different components of MetS, there is a growing interest in establishing miRNA-based therapeutics [164,165]. miRNA therapeutics can be either in the form of miRNA mimics that amplify the impact of a specific depressed miRNA, or antagomiRs that suppress a specific overexpressed miRNA expression. To date, several miRNAs are in different phases of preclinical and clinical trials to be classified as novel therapeutics for several diseases [166,167]. An example of an miRNA-based therapeutic for cardio-metabolic diseases is MGN-9103, a locked nucleic acid (LNA)-modified antisense oligonucleotide (ASO) by Viridian Therapeutics, formerly known as miRagen Therapeutics [168]. MGN-9103 is an antagomiR designed against miR-208, a cardiac-specific miRNA with known benefits for cardiac function and has a therapeutic potential in improving systemic insulin sensitivity and glucose tolerance that contribute to MetS. Another antagomiR includes anti-miR-33, developed by Regulus Therapeutics, which is currently in preclinical stages of development. The use of anti-miR-33 on atherosclerosis regression in diabetic mice was shown to overcome the deleterious effects of T2DM [169].

Although the development of miRNA-based therapeutics could represent a novel treatment approach for various diseases, several challenges remain, including their stability and mode of delivery [170,171]. Commercially prepared miRNA molecules are quite unstable, therefore natural and chemically modified molecules such as 2′-O-methyl (2′-OMe) or LNA are used to stabilize and reduce their high reactivity [170]. Secondly, accurate delivery of these therapeutics to the desired tissue for treatment specificity remains a challenge. Approaches undertaken for the precise delivery of these miRNA therapeutics involve using designed nanoparticles or liposome-like particles incorporating miRNAs that can be targeted to different organs, or combining miRNA with a specific molecule that will bind to the cells of interest and enhance endocytosis [168].

#### 4.4.2. miRNA Biomarkers

Emerging research into the identification of reliable and sensitive biomarkers for the progression and development of different diseases have supported the use of circulatory miRNAs [52,172]. Any deviation from normal miRNA-mediated regulatory networks appear to be a common characteristic of various disease pathogenesis, including cancer, CVDs and other metabolic diseases, thus suggesting circulatory miRNAs could be essential components in the disease pathobiology [173,174]. Circulatory miRNAs in clinical samples are reported to be highly stable, and they can withstand unfavourable physiological conditions, such as variations in pH and storage [175]. Moreover, the diagnosis of diseases with similar aetiologies remains a significant challenge [176]. The abundance of circulatory miRNAs is reproducible with disease and tissue specificity [177]. Since miRNAs meet most of the required criteria for being a biomarker, including accessibility, high specificity and sensitivity, there is an increasing interest in the utility of circulatory miRNAs as potential biomarkers for diagnosis, as well as markers of disease progression [178]. It has to be also highlighted that the therapeutical modulation of distinct miRNAs is gaining importance for the management of patients with MetS features, particularly for T2DM; for instance, it has been recently shown that novel anti-diabetic agents may exert a direct epigenetic effect in T2DM patients, regulating miRNAs involved in the maintenance of endothelial cell homeostasis, and that this effect is independent of the metabolic control [179].

Although several diagnostic measures, including body mass index (BMI), total body fat and blood levels of glucose, HbA1c, lipids and pro-inflammatory cytokines, are available as markers of metabolic health, diagnosis is usually missed in the early stages of these diseases as these markers are generally only detectable upon disease progression [180]. On the other hand, miRNAs have gained increasing attention as a possible means to provide insights into the complex gene regulatory mechanisms involved both in normal physiology and in pathophysiological processes by acting as mediators (and markers) of disease [181]. It has also been reported that variations in miRNA expression profiles can be seen as the body’s integrative response to genetic susceptibility and environmental effects, hence with potential as predictive, diagnostic and prognostic markers [182]. While studies performed both in in vitro and in vivo experimental models have highlighted the associations between altered circulatory miRNA abundances and the dysregulation of contributory factors related to cardio-metabolic diseases [183,184], little is known about the relationship between circulatory miRNAs and the early stages of MetS prior to the development of overt T2DM or CVD.

In relation to miRNAs as biomarkers of MetS, we have previously reported the circulatory miRNAs miR-15a-5p and miR-17-5p as predictive biomarkers of MetS [4]. Both of these miRNAs have an identified role in regulating molecular mechanisms underlying the pathophysiology of metabolic derangements, including β-cell apoptosis, IR and central obesity, which is well known [185,186]. We also observed miR-15a-5p and miR-17-5p abundance to significantly correlate with individual MetS components, including BMI, waist circumference, plasma HDL, plasma glucose and blood pressure. Since MetS represents a complex pathophysiology, correlations of these miRNAs with more than one component suggest that the identified miRNAs might be involved in regulating complex metabolic pathways responsible for the development and progression of MetS. Further, it has been identified that different miRNAs can regulate different components of MetS (Table 3) [187]. One such example would include an increased abundance of miR-221 and let-7g in the circulation of an Asian cohort with MetS [187]. The abundance of both miRNAs was observed to increase with an increasing number of MetS components presented, with let-7g and miR-221 abundance increasing in individuals with more than four components of MetS.

Nonetheless, research into the use of miRNAs as biomarkers for MetS is still in its infancy. To date, the findings generally lack reproducibility, with examples of inconsistent reporting across groups that have analysed the same disease pathology [204,205]. Some of this discordance can be overcome by the use of standardized protocols controlling for both pre-analytical conditions, including sample handling (collection, transport and storage), population under investigation (healthy or diseased) and analytical factors including abundance quantification platforms involving qPCR, high throughput sequencing (HTS), microarray and data normalization strategies (endogenous and exogenous normalizers).

## 5. Conclusions

Considering the metabolic diversity of MetS, together, both classical and miRNA-regulated epigenetic modifications likely act as determining factors for its underlying pathophysiology, and thus may constitute another level of regulation in mediating disease risk. While studies have implicated putative roles for DNA methylation, histone modifications and miRNA regulation on the pathophysiology of MetS, there remains a gap to determine the cause-and-effect relationship between these epigenetic mechanisms and MetS. Therefore, future studies on the dysregulation of the classical miRNA epigenetics machinery in MetS would enable a deeper understanding of MetS pathophysiology. From a therapeutic standpoint, both classical and miRNA-based epigenetic drugs and diets are, in this regard, a flourishing scope for future research directions in the setting of MetS.

## Figures and Tables

**Table 2:** Human studies showing associations between histone modifications and features of metabolic health.

Condition	Histone Modification	Sample Type	Ref
Insulin resistance and inflammation	HDAC3	Peripheral blood mononuclear cells	[43]
Glucose metabolism	HDAC9	Liver	[48]
Vascular dysfunction and T2DM	Set7	Peripheral blood mononuclear cells	[49]
Obesity and T2DM	SIRT1	Visceral adipose tissue	[46]
Glucose metabolism	Swi/Snf	Pancreatic islet beta cells	[50]

**Table 3:** Human studies reporting association between circulatory miRNAs and features of metabolic health.

Disease	miRNA	Sample Type	Ref
MetS/Atherosclerosis/Obesity	miR-15a-5p	↓ Plasma	[4,188]
MetS/Obesity	miR-17-5p	↓ Serum/Plasma	[4,189]
MetS Chronic Heart Disease with T2DM	miR-21-3p	↓ Plasma ↑ Plasma	[190]
Obesity/Chronic Heart Disease	miR-29a-5p	↑ Plasma	[191]
T2DM	let-7 family	↑ Plasma	[192]
Obesity/T2DM	miR-122-5p	↑ Plasma	[193]
Atherosclerosis/T2DM	miR-126-5p	↓ Plasma	[194]
Obesity	miR-143-5p	↓ Plasma	[195]
T2DM	miR-144-5p	↑ Plasma	[196]
Obesity/T2DM	miR-221-3p	↓ Plasma	[197]
Obesity	miR-222-3p	↑ Plasma	[198]
T2DM/Obesity	miR-320a	↑ Plasma	[199]
T2DM/Diabetic Cardiomyopathy	miR-370-3p	↑ Plasma	[200]
T2DM	miR-375	↑ Serum	[201]
Diabetic Cardiomyopathy	miR-186-5p	↑ Plasma	[202,203]

↓ indicates decreased abundance compared to controls; ↑ indicates increased abundance compared to controls.
